# Observations on the Theory of Fever

**Published:** 1812-10

**Authors:** 


*To the Editors of the Medical and Physical Journal.*

GENTLEMEN,

I BEG leave to acquaint you that I have practised physic for upwards of twenty years, and during that period I have been in almost every variety of climate. Now having retired from business, I mean from time to time to make you acquainted with some of my theoretical opinions, as well as practical observations, and give you full liberty to insert them in your valuable periodical publication, provided you think proper.

About twelve years ago, I first perused the invaluable work of the immortal Currie, of which I am an enthusiastic admirer, not only as to his practical observations, which are certainly founded on the solid basis of sound judgment, and supported by accurate observation and nice discrimination; but I also equally admire his theoretical opinions of fever, which last are, in my opinion, however, liable to some objections.

Dr. Currie attributes the reaction in fever chiefly to the impulse given to the heart and arteries by the determination of blood which takes place towards the centre of the system during the cold stage. If this really were the case, an unusually increased action of the heart and arteries should exist for some time before a preternatural increase of temperature takes place. But, as a preternatural increase of temperature occurs in every instance (as far as my observation goes) before an increased vigor of the circulation comes on, it seems to me perfectly reasonable to infer that the unusual increase of heat is the cause of the increased impetus of the blood.

Indeed it appears, from Dr. Currie's own experiments, that, when the temperature of a person laboring under fever is reduced to the natural standard by the affusion of cold water, that the pulse is weaker than natural; an undoubted proof to me that the increased vigor of the circulation depends upon upon the preternatural increase of temperature, and that the essence of fever is diminished nervous energy; and, granting this to be the case, I think that all the phenomena of fever may be satisfactorily accounted for, and are only to be regarded as the consequences of diminished nervous energy.

If fever consists in diminished nervous energy, it may be asked, how is it possible that the health of a person is restored after laboring under fever for several weeks, and being at length reduced to extreme debility; and more especially if exposed at the same time to the remote causes which originally gave rise to the disease, as, for instance, in an hospital exposed to the effluvia of febrile patients. This, at first sight, I own is apparently a plausible objection to the doctrine now advanced; however, with a little reflection, the mystery may in some measure be cleared up.

In the commencement of fever, the debility arises from diminished nervous energy; but, when the fever goes off, the energy of the nervous system is restored, and the extreme debility that then often exists is owing to want of muscular strength, the muscular substance being gradually wasted in the course of the disease, consequently general debility must exist to a greater or less degree, until the muscles of the whole body are restored to their wonted vigor. But the mental powers are generally fully restored as soon as the fever ceases; hence the debility existing at the commencement of fever, is totally different from that which exists after the fever has gone off, the former arising from diminished nervous energy, and the latter from the waste of muscular substance throughout the whole system.

How the energy of the nervous system is restored under such circumstances, I must confess that I am entirely ignorant. During the first stages of fever, the sudden cures performed by cold bathing sufficiently prove that the disease then depends entirely on a peculiar debilitated state of the nervous system; but, as in the advanced stages of fever, the cure is generally effected very gradually, whatever means may be used, I think it must be granted that the removal of the disease depends principally on some powerful change which gradually takes place in the nervous system, which, in many instances, is not only sufficient to restore the energy of the brain, but also at the same time enables the system to resist the noxious influence of the remote causes, which may originally have occasioned the disease. Is it possible that, during fever, the blood undergoes a certain change, which, operating on the nervous system, is capable of restoring the energy of the brain?

As to the success attending the affusion of cold water in fevers, fevers, it must have excited the attention and admiration of every one who has read Dr. Currie's work. When lately reading the last edition, I was forcibly struck with the uncommon success of the practice of Mr. Nagle, formerly surgeon of his Majesty's ship Ganges, 74, while on the Jamaica station in 1801 and 1802.

When the Ganges arrived at Port Royal harbour, Jamaica, in November 1801, the crew were remarkably healthy; however, Mr. Nagle says, "We found a malignant fever prevailing among the shipping there. The mortality was particularly great on-board the merchant ships at Kingston, many of them being almost unmanned by it. Soon after our arrival it broke out on-board the Ganges, and spread rapidly, especially among the marine and landsmen who had never before been in a warm climate. The symptoms were severe head-ach, hot and dry skin, the face flushed, eyes red, nausea, thirst, pulse strong and full at first and as frequent as 120 in a minute, pains in the back and limbs, great anxiety and restlessness. The patients were generally under much depression of spirits, from the account we had received of the great mortality of the fever. There was little chilliness in any stage of the disease, and remissions were scarcely perceptible. Heat of the skin was the most striking symptom.

"The violent and rapid nature of the disease, convinced me that early and decisive measures were required, and I determined to have recourse to the affusion of cold water, under the directions which you have given for its use.

"As soon, therefore, as the morbid heat fairly indicated the accession of fever, I poured a quantity of sea-water on the patient, from the head downwards, generally two or three buckets full, and commonly directed the body to be afterwards wiped with a towel dipped in vinegar, but more with a view of preventing the sailors from thinking that I trusted entirely to the cold water, than from any supposition of the vinegar being required. I then put the patient into bed, gave him in general from eight to ten grains of calomel with four or five grains of the pulv. antimonial, and, supplying him with plenty of diluent drink, left him to his repose.

"The affusion, when used on the second or third day of fever, operated like a charm. The morbid heat and dryness of the skin was converted into an agreeable coolness with some degree of moisture, the pulse sunk very often from 120 to 90, the head-ach, flushings, restlessness, and agitation, disappeared, sensations of comfort were diffused over the whole body, and the patient fell into a natural and refreshing sleep. On waking, two or three passages downwards from from the calomel seemed to carry off any remaining irritation. Most commonly the fever did not return, but, if it did, the bathing was repeated once, or perhaps twice, as might be required. Where I had not an opportunity of seeing the men for the first day or two of fever, which sometimes happened from their being taken ill on shore and remaining there, the effects of the affusion, though strikingly beneficial, were not so immediately decisive, and it was requisite to repeat it several times. I had seldom occasion to use opium in this fever, for the cold affusion produced sleep, and, in three or four cases in which I gave opiates at bed-time, irritation and restlessness ensued, the symptoms being increased, which the cold affusion had obviated. We had one hundred and twenty cases of fever in all, during the time which I served on-board the Ganges on the Jamaica station, that is, from November 1801, to the end of July 1802, in all of which the cold affusion was used, and of which we lost two only. One of these had been ill of a violent inflammation in the knee, for which I was obliged to use bleeding largely, and in this reduced state he was attacked with fever; the other was a marine, of a weakly habit and of a consumptive tendency."

The success of Mr. Nagle's practice is no doubt very remarkable, and is attributed by him, and likewise by Dr. Currie, almost entirely to the affusion of cold water.

Far be it from me to derogate any thing from the justly high repute which cold bathing has acquired in febrile diseases. On the contrary I hold it in the highest estimation, and consider it the most powerful and efficacious remedy hitherto known, when repeated as often as the system acquires a preternatural increase of temperature. However, I cannot help imputing Mr. Nagle's success in a considerable degree to the powerful febrifuge effects of a combination of calomel and antimonial powder.

For these ten years I have generally been accustomed to prescribe, towards the commencement of febrile diseases, a composition of calomel and James's powder, and assert, that in most instances, when the medicine was prescribed early in the disease, that an intermission or a very considerable remission took place in less than twenty-four hours after its exhibition, provided a copious perspiration ensued. My patient's drink generally consisted of barley or rice water mixed with wine and sugar, or weak broths occasionally. Decoction or infusion of bark were prescribed as soon as an intermission or a very considerable remission took place, and in most cases the fever did not return. In plethoric persons I generally premised blood-letting.

 I consider

I consider it a duty incumbent on me to state, that I learned this practice from a small publication of Mr. Thomas Clark, formerly surgeon of the 17th regiment of foot, published at Edinburgh in 1801. And, with a view to point out as clearly as possible the extraordinary febrifuge powers of a composition of calomel and James's powder, I shall give an abridged account of Mr. Clarke's practice, for I believe his small, though valuable, work is as yet very little known.

Mr. Clark says that he first began the early administration of calomel and James's powder in fevers in the latter end of 1795, and that in every case of fever, previous to the exhibition of the medicine alluded to, he bled his patients moderately, with a view to relieve the general plethora occasioned by the increase of temperature, and thereby rendered the operation of sudorifics safe.

He embarked in the Britannia East-Indiaman in the latter end of October, 1796, with upwards of four hundred men, women, and children; and, after having been on-board for nearly four months, and having made two unsuccessful essays to get to the West Indies, he had the satisfaction to land his crew without the loss of a man, notwithstanding the number of sick on-board the Britannia had been very considerable; and he only sent two men to an hospital ship about a week before the detachment of the 19th regiment disembarked, both of whom afterwards joined the regiment.

Mr. Clark was forcibly struck with the success attending this practice, and, previous to the 19th regiment embarking for India in May following, he made his assistants intimately acquainted with the practice alluded to.

The regiment embarked on-board three ships, and the Ocean, on which he himself was on-board, stopped at the Cape of Good Hope for about six weeks. The other two ships proceeded direct for India, the one for Madras, and the other for Bombay. On-board the Ocean, during a period of about six months, three consumptive patients died, one of whom was ill when he left England, and one man died in consequence of an accident; on-board the ship bound for Bombay one man died only; and on-board that for Madras nobody died. The transporting of a regiment to India nearly eleven hundred strong, with so trifling a loss, is, I believe, a very rare occurrence; consequently I cannot help attributing this uncommon success, in a great measure, to the decisive mode of practice adopted by Mr. Clark.

From Mr. Clark's abstract of sick while in India from the 10th of December, 1796, to the 1st of November, 1798, it appears that he had seven hundred and eleven patients laboring boring boring under fever admitted into his hospital, and that only seven of these died. Such strong facts speak for themselves.

For a considerable length of time after Mr. Clark's arrival in India, he bled his patients, in almost every instance, previous to the administration of the sudorific medicine; but afterwards he only bled in the more violent cases, the symptoms of plethora being less frequent.

Hence, I think, it must be granted that Mr. Clark's practice has been attended with great success, and consequently it must be allowed that combinations of calomel and James's powder administered early in fevers, may frequently cut short the disease.

In most of the cases treated by Mr. Nagle, it was unnecessary to repeat the affusion of cold water even a second time; but, according to the accounts given by other West India practitioners concerning the effects of cold bathing in fevers, it has generally been found requisite to use the cold affusion frequently, and their success, upon the whole, has been far inferior to that of Mr. Nagle; an undoubted proof, in my opinion, that much of Mr. Nagle's success is to be attributed to the effects of calomel and antimonial powder.

By Dr. Currie's reports it uniformly appears that permanent advantage has only been derived from cold affusion when it was succeeded by sweating; consequently, cold bathing in fevers may, in a great measure, be regarded as a sudorific medicine; and hence the great benefit obtained by employing the two powerful remedies alluded to at the same time.

Thus it would appear that we are possessed of two very powerful febrifuge remedies, namely, the affusion of cold water, and a combination of calomel and antimony; would it not, therefore, be adviseable to use both these agents at the same time in all violent febrile affections, as was done by Mr. Nagle, and in cases of unusual plethora, or where local inflammatory affections existed; might it not also be adviseable to premise bleeding? Whatever may be said against blood-letting in fevers, I am fully convinced, from my own practice, that, when had recourse to in the beginning of the disease, it is often beneficial; but, in the more advanced stages of fever, I believe it to be highly injurious; at the same time it appears to me extremely probable that the cold affusion, previous to the exhibition of the sudorific, may in most instances supersede the necessity of bleeding.

*An impartial Medical Man.*

*London, August* 11, 1812.

